# The Charity Organisation Society and the Hospitals

**Published:** 1897-06-05

**Authors:** 


### Editor's Letter-Box.

[Our Correspondents are reminded that prolixity is a great bar to publication, and that brevity of style and conciseness of statement greatly facilitate early insertion.]

### THE CHARITY ORGANISATION SOCIETY AND THE HOSPITALS.

Mr. C. S. Loch, Secretary of the Charity Organisation Society, writes: I am desired to point out two very grave, and I think I may add almost unpardonable, misstatements in the article published in your issue of the 22nd ult., entitled, "The Charity Organisation Society and the Hospitals." First, neither has the Charity Organisation Society nor have I myself directly or indirectly endeavoured, as you state, to make the Prince of Wales's London Hospitals Fund unsuccessful. On the contrary, we have wished the fund every possible success, only urging that for its administration a board might with advantage be formed, which included amongst its members lay and medical representatives of the medical charities of the metropolis. Next, neither has the society nor have I myself directly or indirectly opposed the voluntary system of hospitals. The absolute reverse is the fact. It must be a matter of regret to all who care first and foremost for an honourable and impartial consideration of such questions that in a controversy on the important subject of the formation of a Central Hospital Board for London, you should think it right to use such misstatements as these as a means of influencing your readers.

*** First, has Mr. Loch endeavoured to make the Prince of Wales's London Hospitals Fund unsuccessful? We take it that anything which throws doubt upon its desirability or utility or upon its being properly distributed must make people hesitate about contributing to it, and so in the most positive manner make it unsuccessful. Turning to Mr. Loch's letter to the *Times*, dated February 11th, we find him urging the necessity for what he terms "safeguards" in regard to the use of the Fund, and so far is he from admitting that such safeguards exist that, he says, in regard to the proposal to rely on the co-operation of the Hospital Sunday Fund, "but this suggests rather alarm than confidence." Is not this calculated to make people question whether the Fund is arranged on a sound basis, and doubt as to the desirability of giving their money to it? Again, he says, "I would ask for an explicit assurance that His Royal Highness's Fund will not be used to subsidize out-patient or casualty department, either directly or indirectly." Now, when a gentleman occupying the position which Mr. Loch does, and writing from the office of a body established with the object of organising the charity of the whole country, writes publicly asking for "an explicit assurance," and guarding the subject of this assurance by the term, "directly or indirectly," every reader must believe that unless and until such an assurance is given Mr. Loch, wielding such influence as he does, is opposed to the fund. Can anyone believe that money can be ear-marked, and that assistance given to any hospital in London can be prevented from "directly or indirectly" helping its out-patient department. Yet, according to Mr. Loch, if they do so help they will be doing the very thing that Mr. Loch demands "explicit assurances" that they shall not do, or, in Mr. Loch's words, they will be "making their fellow-citizens by their charity more dependent, or, in other words, less competent to fulfil the duties of life and to bear its responsibilities." How many thousands of possible givers there must be who on hearing this note of warning sounded by Mr. Loch have buttoned up their pockets and declined to give to a fund on which he has thrown such doubt! As to the second point. Does Mr. Loch, or does his society, oppose the voluntary system of hospital support? We do not think that Mr. Loch will agree with us in this matter, but we consider that the attempt to subordinate the hospital boards, which have been elected by the voluntary subscribers, to a central board, such as has been proposed under the sanction of, if not actually by, the Charity Organisation Society, would be disastrous to the voluntary system; and by the part he has played in forwarding these proposals we maintain that Mr. Loch has very definitely opposed the voluntary system of hospital support; for this includes the management of the hospitals as well as their maintenance by those who subscribe to their funds.—Ed. *T.H.*

